# Risk of hematological malignancies and anticipation in the families of patients with non-Hodgkin and Hodgkin lymphoma

**DOI:** 10.3389/fonc.2025.1639819

**Published:** 2025-09-09

**Authors:** Saad Akhtar, Ali Hassan Mushtaq, Huda Syed, Tusneem Ahmed M. Elhassan, Meshari Jamaan Alzahrani, M Shahzad Rauf, Mahmoud A Elshenawy, Irfan Maghfoor

**Affiliations:** ^1^ King Faisal Specialist Hospital and Research Centre, Oncology Center, Riyadh, Saudi Arabia; ^2^ College of Medicine, Alfaisal University, Riyadh, Saudi Arabia; ^3^ Clinical Oncology Department, Faculty of Medicine, Menoufia University, Shebin El Kom, Egypt

**Keywords:** familial lymphoma, non-Hodgkin lymphoma, Hodgkin lymphoma, familial clustering in lymphoma, familial clustering of malignant neoplasms, anticipation

## Abstract

**Objectives:**

Data on patients with non-Hodgkin lymphoma (NHL) and Hodgkin lymphoma (HL) with a family history of malignancy (FHM) and anticipation are limited. These patients provide an opportunity to study the responsible genes and clinical outcomes. There is no comprehensive report from the Middle East, where large family sizes and consanguineous marriages are common. Here, we report our observations.

**Methods:**

This was a retrospective, single-institution cohort study. Patients seen in the lymphoma clinics were comprehensively interviewed for FHM.

**Results:**

We interviewed 1,274 lymphoma patients for FHM: 591 (46.4%) with NHL and 683 (53.6%) with HL; 745 (58.5%) were men. The median age was 32 years. Consanguineous marriages were reported in 9.1% (parents) and 9.2% (patients). Among them, 524 (41.1%) patients had no FHM, whereas 750 (58.9%) had FHM. Patients reported a total of first-, second-, and third-degree relatives and 1,249 relatives with 1,263 malignancies. In total, 254 patients reported family members with hematological malignancy only (131, 10.3%) or hematological plus solid malignancies (123, 9.7%), while 496 (38.9%) reported only solid cancers. In total, 254 patients identified 305 affected relatives: NHL, 67 (22%); HL, 70 (23%); lymphoma not otherwise specified (NOS), 30 (9.8%); leukemia, 111 (36.4%); and other hematological malignancies, 27 (8.5%). Relationship pairs (n = 305) included parent/child (36), sibling/sibling (54), sibling/half-sibling (6), uncle/aunt or nephew/niece (46), grandparent/grandchild (14), and patient/cousin (59). These 305 disease pairs were: NHL/NHL, 39 (12.8%); NHL/lymphoma NOS, 17 (5.6%); NHL/HL, 66 (21.6%); NHL/leukemia, 47 (15.4%); NHL/others, 4 (1.3%); HL/HL, 51 (16.7%); HL/lymphoma NOS, 13 (4.3%); HL/leukemia, 64 (21%); and HL/others, 4 (1.3%). Anticipation data were available for 92 pairs (63.3%); earlier age at diagnosis in the first generation (58 years) versus the second generation (24 years, p=<no><</no> 0.001) was significant, even after correction for ascertainment bias.

**Conclusion:**

In patients with NHL and HL, FHM is common, and anticipation was observed. Future studies should explore the genetic basis of these findings.

## Introduction

Emerging data indicate an increased risk of various lymphoid and hematological malignancies in family members of patients with non-Hodgkin lymphoma (NHL) and Hodgkin lymphoma (HL), suggesting a hereditary component in this population. Familial hematological/lymphoid malignancies (FHM), such as NHL, HL, nodular lymphocyte-predominant Hodgkin lymphoma (NLPHL), multiple myeloma (MM), leukemia, and chronic lymphocytic leukemia/small lymphocytic lymphoma (CLL/SLL), have been reported to occur in clusters within families (1–12). Patients and families with FHM provide an opportunity to study and identify genetic, environmental, and triggering causative factors. Most FHM data come from registry-based studies in North America and Europe, where family sizes tend to be smaller (1, 3, 4, 8, 11). In contrast, Middle Eastern populations have distinct social structures, including tribal lifestyles, intra-tribal marriages, larger family sizes, cultural and environmental factors, and a high prevalence of consanguineous marriages. The impact of these factors on FHM remains largely unknown and may vary from Western populations.

Anticipation is defined as an increase in disease severity or a decrease in age at diagnosis between successive generations, and it has been described in many malignancies, including hematological/lymphoid cancers (7–9, 12–18). The present study aims to identify FHM in patients with NHL and HL who presented to our medical oncology lymphoma clinics and to establish a hospital-based FHM database. This pilot effort will support future initiatives in data collection, genetic counseling/studies, and tissue banking. We also aim to evaluate the anticipation phenomenon and apply methods to correct for ascertainment bias (7, 9, 14, 17).

## Methods

This data collection study was approved by the Institutional Research Advisory Council and Ethics Committee (prospective/retrospective lymphoma data collection; project and publication approval numbers 2021048 and 2245276, respectively), along with the supplemental questionnaire (Arabic and English) of family history of malignancy (FM) case report form (FM-CRF) (ClinicalTrials.gov Identifier: NCT00538551). These resources were designed to capture all solid and hematological malignancies in first-, second-, and third-degree relatives, consanguineous marriages, age at diagnosis in family members, half-siblings (not uncommon in this population), and survival status, as reported previously (12). The Arabic version of the questionnaire was translated by three native Saudi Arabic–speaking oncology nurses with the assistance of patients. It was administered in person to patients and/or their parents or caregivers during routine outpatient clinic visits and was followed up by phone when needed. The detailed methodology has been described previously (12). Patients seen in the lymphoma clinics from 2015 to 2019 were interviewed, with a limited number interviewed before that period. All questions were asked, and answers were recorded regardless of the initial response. Relatives treated at our institution were verified using hospital medical records and/or the Hospital Tumor Registry (data from 1974 - 2020). These patients and their relatives were carefully re-reviewed to avoid duplication. The degree of relationship was defined as follows: 1st-degree relatives (parents, siblings, or children), 2nd-degree relatives (grandparents, grandchildren, or half-sibling (stepbrother/stepsister), aunt, uncle, nephew, or niece), and 3rd-degree relatives (first cousins, great-grandparents, or great-grandchildren). An FM is considered “confirmed” if one of the following criteria was met; (a) a pathology report, (b) a medical report with diagnosis, (c) a reliable history with confirmation of malignant disease by a healthcare professional, or (d) confirmation from The Saudi Cancer Registry (a population-based registry in the Ministry of Health with a site office in our Research Unit, data available from January 1, 1994 – 2015). If criteria a–d were not met, the patient was considered an “unconfirmed case,” provided that the interviewing physician deemed the history sufficient to classify it as a malignancy. Due to time and effort constraints, most confirmed malignancies were limited to FHM.

### Statistical analysis

The Statistical Package for the Social Sciences (SPSS), version 17, was used to analyze frequencies, medians, and percentages. The chi-square test was applied for frequency analysis. Hazard ratios (HRs) with confidence intervals (CIs) were calculated using linear regression to assess significance.

Our institution is a tertiary care referral center; therefore, our patients do not represent a population-based collection. As such, we could not estimate a population size to use as a denominator for calculating the incidence of lymphoid and hematological malignancies in families compared with the general population.

Anticipation studies: The null hypothesis assumed that the age at diagnosis of hematological and lymphoid malignancies would be the same in first and second generations. As reported in similar studies, to evaluate the effects of ascertainment bias, all cases diagnosed before the age of 25 years were excluded, and statistical analysis for anticipation was repeated (7, 9, 14, 17).

## Results

### Overall results of the entire cohort

A total of 1,274 patients completed the FH-CRF. From January 2015 to July 2019, 2,507 patients with NHL and HL accounted for 17,810 visits to the outpatient lymphoma clinics of three physicians (SA, IM, and MSR). During this period, 1,189 of 2,507 patients (47.4%) were given the questionnaire, and 1,094 of 1,189 (92%) responded and completed the FH-CRF. Another 180 patients (14.1%) had completed it before 2015.

Of the 1,274 patients, 745 (58.5%) were men and 529 (41.5%) were women; 683 (53.6%) had HL and 591 (46.4%) had NHL. The median age at diagnosis for the entire group was 34 years (range, 8 – 99).

**Figure 1:**
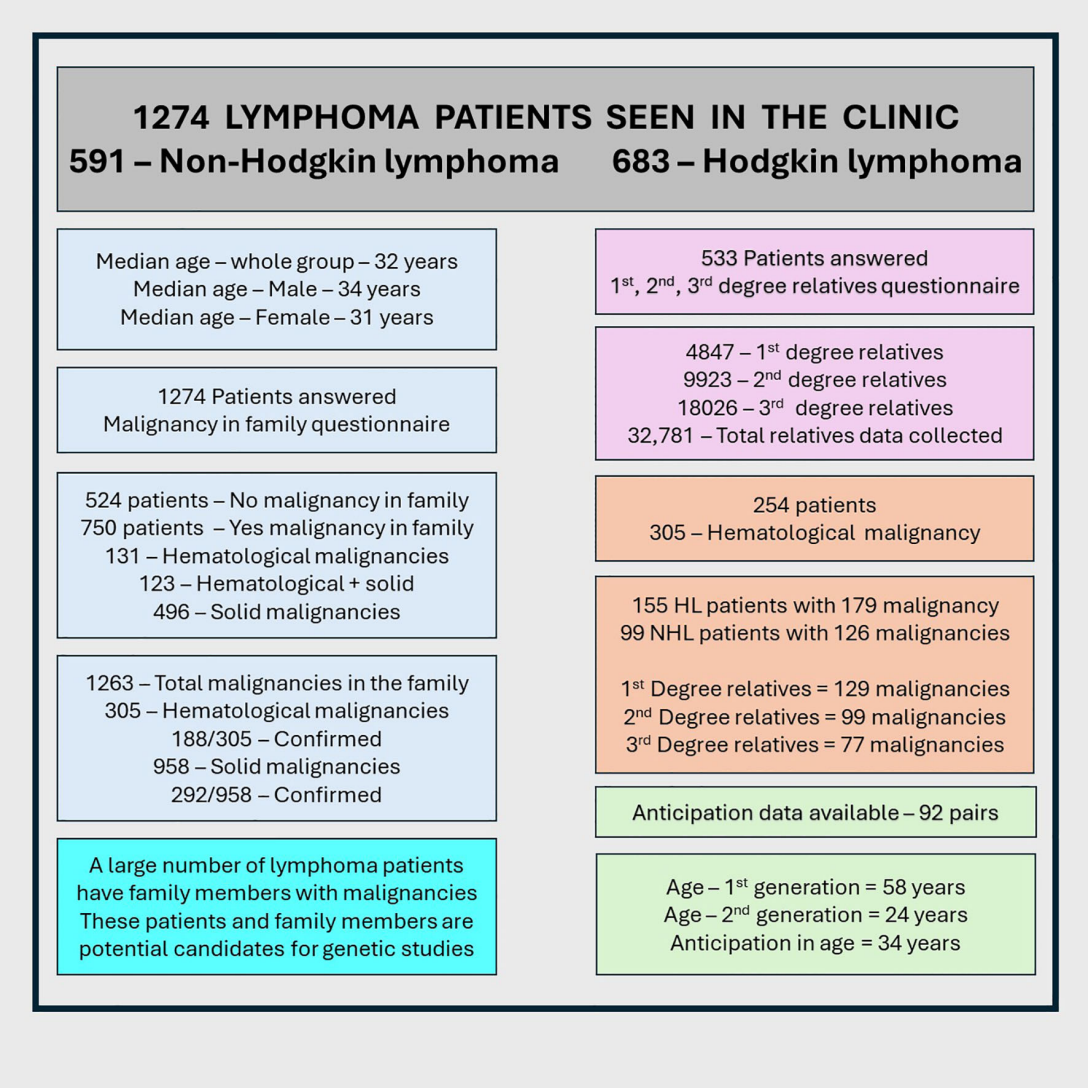
Diagrammatic flow representation of the results.

Consanguineous marriage was reported in 117 patients (9.2%) (missing data, 719 [56.4%]) and in 116 of their parents (9.1%) (missing data, 942 [73.9%]). For family size, 533 of 1,274 patients responded, providing information on a total of 32,781 first-, second-, and third-degree relatives (**Table 1**, **Figure 1**).

**Table 1:** Patient’s characteristics and family size.

Variable	Total numbers	Percentage
Total patients completing interview	1,274	100
Interviewed from 2015 - 2019	1,094	85.9
Interviewed prior to 2015	180	14.1
Male patients	745	58.5
Female patients	529	41.5
Median age (whole group)	32 (8 - 99)	–
Median age (Male patients)	34 (8 - 99)	–
Median age (Female patients)	31 (8 - 86)	–
Age <21 years	304	23.9
Age >21–30 years	284	22.3
Age >30–50 years	391	30.7
Age >51 years	295	23.2
Consanguinity information (patients)		
Yes	117	9.2
No	189	14.8
Not applicable	249	19.5
Unknown	719	56.4
Consanguinity information (parents)		
Yes	116	9.1
No	216	17
Unknown	942	73.9
Information available for first-, second-, or third-degree relatives	533	41.8
First-degree (families) reported	529	41.5
Total first-degree relatives reported (range)	4,847 (2 - 22)	–
Second degree (families) reported	509 (4 - 72)	40
Total second-degree relative reported (range)	9,923 (4 - 200)	–
Third-degree (families) reported	435	34.1
Total Third-degree relative reported (range)	18,026 (3 - 245)	–
All first-, second-, and third-degree relatives reported	32,781	3.85% with cancer -

We also analyzed all parents with consanguinity and patients older than 40 years to examine its impact on family history of malignancies. Among those with consanguinity, 69 of 150 (46%) had a family history of cancer compared with 103 of 196 (52.6%) without consanguinity (p = 0.27).

For first-degree relatives, patients reported a median of 9 relatives (range, 2 – 22); for second-degree relatives, 18 (range, 4 – 72); and for third-degree relatives, 34 (range, 3 – 245) (**Table 1**). Based on a median family size of 61, approximately 75,000 – 80,000 total family members’ data would have been captured if all responses were available. 

No family malignancy was reported in 524 patients (41.1%), whereas 750 patients (58.9%) reported malignancies in their families. Hematological malignancies were reported in 254 patients (19.6%), including 123 (9.7%) who also had solid cancers; 131 (10.3%) had hematological malignancy only. Solid cancers alone were reported in 496 patients (38.9%).

Across all 1,274 patients, 1,249 family members were reported with a total of 1,263 malignancies: 305 (24.1%) hematological and 958 (75.8%) solid cancers. Of these 1,263 malignancies, 480 (37.7%) were confirmed, comprising 188 hematological (14.9%) and 292 solid (23.1%) cancers. Among the 305 hematological malignancies, 188 were confirmed (61.6%).

Among 958 solid cancers, the most common were breast cancer (173, 18.1%), gastrointestinal cancers (102, 10.6%), colorectal cancers (97, 10.1%), and head and neck cancers (70, 7.3%); other cancers are shown in **Table 2**.

**Table 2:** Patient and family member diagnoses.

Variable	Total numbers	Percentage
Diagnosis in patients		
Classical Hodgkin lymphoma	536	42.1
NLPHL	147	11.5
DLBCL	379	29.7
NHL – low grade, follicular, NOS	176	13.8
NHL – T cell lymphomas	36	2.8
Family member characteristics		
Families with no malignancy	524	41.1
Families with any malignancy	750	58.9
Families with hematological only	131	10.3
Families with hematological + solid	123	9.7
Families with solid cancers only	496	38.9
Total members with malignancy	1249	–
Total malignancies in family members*	1263	100
Hematological malignancies	305	24.1
Malignancy confirmed	188	14.9
Malignancy confirmed – of 305	188	61.6
Malignancy unconfirmed	117	9.3
Solid malignancies	958	75.8
Malignancy confirmed	292	23.1
Malignancy confirmed – of 958	292	30.5
Malignancy unconfirmed	666	52.7
NHL patients (591) and total malignancies	343	58
HL patients (683) and total malignancies	407	59.6
Common familial malignancies in the family members - by groups		
Hematological malignancy	305	100
Non-Hodgkin lymphoma – NOS	37	12.1
DLBCL	30	9.8
Hodgkin lymphoma	70	23
NHL/HL (Lymphoma NOS)	30	9.8
Leukemia	111	36.4
Others/Some hematological NOS	27	8.5
Solid Cancers	958	100
Breast	173	18.1
NOS	135	14.1
Gastrointestinal (esophagus, stomach, pancreas, gallbladder	102	10.6
Colorectal Cancer	97	10.1
Head and Neck	70	7.3
Brain	64	6.7
Liver	60	6.3
Lung	58	6.1
Female Genital	56	5.8
Bladder and kidney	41	4.3
Thyroid	40	4.2
Prostate	25	2.6
Others	37	3.9

*Fourteen family members had two malignancies each; counts were also adjusted accordingly.

Of the 1,274 patients (591 with NHL and 683 with HL), 750 had one or more family members with malignancies (solid or hematological). In patients with NHL, 343 (58.2%) reported family members with malignancy compared with 407 (59.4%) among patients with HL (p = 0.67; HR = 0.95, 95% CI: 0.71 – 1.20).

Among the 254 patients with family members affected by hematological malignancies, 99 (16.8%) had NHL and 155 (22.6%) had HL (p = 0.01; HR = 0.69, 95% CI: 0.52–0.91).

Among the 619 patients with family members affected by solid cancers, 293 (49.2%) had NHL and 326 (47.6%) had HL (p = 0.44; HR = 1.10, 95% CI: 0.87 – 1.36) (**Tables 2**, **3**).

**Table 3:** Types of hematological malignancies in the family members of the patients.

Diagnosis – Patients		Diagnosis – Family members					Total
Diagnosis	Number (%)	HL	Leukemia	NHL	Lymphoma NOS	Others	
HL	155 (61)	51 (16.7)	64 (21)	47 (15.4)	13 (4.3)	4 (1.3)	179 (58.7)
NHL	99 (39)	19 (6.2)	47 (15.4)	39 (12.8)	17 (5.6)	4 (1.3)	126 (41.3)
Total	254 (100)	70 (23)	111 (36.4)	86 (28.2)	30 (9.8)	8 (2.6)	305 (100)

Abbreviations as per **Table 1**.

Due to the large NLPHL population, we compared the difference in malignancies between classical HL (536 patients) and NLPHL (147 patients). Among patients with HL, 323 (60%) had family members with a malignancy, compared with 84 (57.1%) patients with NLPHL (P = 0.61; HR = 1.13, 95% CI 0.78 – 1.6). For patients with family members who had a hematological malignancy, 124 (23%) had HL and 31 (21.1%) had NLPHL (P = 0.61; HR = 1.12, 95% CI 0.72 – 1.75). For patients with family members who had a solid cancer, 254 (47.2%) had HL and 72 (49%) had NLPHL (P = 0.7; HR 0.93, 95% CI 0.65–1.34) (**Table 3**).

### Patients with hematological malignancies

A wide spectrum of hematological malignancies was observed among 305 family members of 254 patients: NHL, 35 (11.5%); diffuse large B-cell lymphoma (DLBCL), 30 (9.8%); Burkitt lymphoma, 8 (2.6%); low-grade NHL, 7 (2.3%); small lymphocytic lymphoma, 4 (1.3%); NHL–T cell, 2 (0.6%); lymphoma NHL/HL with no other subclassification available, 30 (9.8%); HL, 62 (20.3%); nodular lymphocyte-predominant HL (NLPHL), 8 (2.6%); leukemia not otherwise specified (NOS), 73 (23.9%); acute myeloid leukemia (AML), 15 (4.9%); acute lymphoblastic leukemia (ALL), 15 (4.9%); other leukemias (including aplastic anemia, CLL, and CML), 8 (2.6%); multiple myeloma, 4 (1.3%); and other malignant blood disorders, 4 (1.3%) (**Tables 2**, **3**).

The co-occurrence patterns of various hematological malignancies were also recorded (**Table 3**). These 305 patient/family member pairs were as follows: NHL/NHL, 39 (12.8%); NHL/lymphoma, 17 (5.6%); NHL/HL, 66 (21.6%); NHL/leukemia, 47 (15.4%); NHL/others, 4 (1.3%); HL/HL, 51 (16.7%); HL/lymphoma, 13 (4.3%); HL/leukemia, 64 (21%); and HL/others, 4 (1.3%).

### Degree and relationship among family members

The 254 patients with hematological malignancies reported a total of 6,186 family members: 807 first-degree, 1,785 second-degree, and 3,594 third-degree relatives. Most patients reported only one family member with a hematological malignancy (215 families, 84.6%), whereas 29 (11.4%), 6 (2.4%), and 2 (0.8%) families reported two, three, and four affected members, respectively.

Among the 807 reported first-degree relatives (both affected and unaffected), 129 had hematological malignancies: 49 were parent/child pairs and 80 were sibling pairs. Other relationships and details are shown in **Table 4**.

**Table 4:** Degree and type of relationship, by the size of hematological malignancies families.

Family members with malignancy	One case	Two cases	≥ Three cases*	All cases
Families	215	29	10	254
Cases	215	58	32	305
Blood relatives				
1°	90	27	12	129
2°	65	25	9	99
3°	60	6	11	77
Relationship				
1° Parent-child	36	9	4	49
1° Siblings	54	18	8	80
2° Aunt/uncle-niece/nephew	47	22	3	71
2° Grandparent-child	14	3	6	23
2° Half-siblings	6	–	–	6
3 °Cousins	58	6	11	75
Gender				
Male	129	36	24	189
Female	81	22	7	110
Unknown	5	–	1	6

*≥ Three cases include eight families with three cases and two families with four cases.

### Anticipation

Anticipation was applicable in 143 pairs; data were available for 92 pairs (63.3%) and missing for 51 pairs (35.6%). These pairs included 38 parent/child (41.3%), 13 grandparent/grandchild (14.1%), and 41 uncle/aunt–nephew/niece (44.6%) relationships.

The median age at diagnosis in the first generation was 58 years overall (NHL, 58 years; HL, 42 years; other malignancies, 59.5 years). The median age for parents was 60 years compared with 32 years for their children (p ≤ 0.001) (**Table 5**). For grandparents, the median age was 71.5 years, and for uncles/aunts, it was 42 years.

**Table 5:** Anticipation in various generations and groups.

Anticipation - Age in years	Median age at diagnosis (years)	The difference in age (years earlier)	*P*-value
Generation/(Patient group) (numbers)			
Generation one (all cases) (88)	58	34	*P*=<0.001
Generation two (all cases) (87)	24		
Generation one (NHL only) (49)	62	31	*P*=<0.001
Generation two (NHL only) (29)	31		
Generation one (HL only) (19)	42	18	*P* = 0.001
Generation two (HL only) (43)	24		
Generation one (Others only) (20)	59.5	45.5	*P*=<0.001
Generation two (Others only)(15)	14		
Parents (38)	61	29	*P*=<0.001
Children (38)	32		
Grandparent (13)	72	50	*P*=<0.001
Grandchild (13)	22		
Uncle/Aunt (41)	42	20	*P*=<0.001
Nephew/Niece (41)	22		
Ascertainment (Age >25 years at Dx)			
Generation one (41)	62	28	*P*=<0.001
Generation two (41)	34		
Generation one (NHL only) (24)	64	30	p=<0.001
Generation two (NHL only) (21)	34		
Generation one (HL only) (4)	56	25	p=<0.001
Generation two (HL only) (19)	31		

143 pairs data applicable.

92 pairs data available = 63.3%.

51 pairs data not available = 35.6%.

The median age at diagnosis in the second generation was 24 years overall (NHL, 31 years; HL, 24 years; other malignancies, 14 years) (**Table 5**). The difference of 34 years in median age between the first (58 years) and second (24 years) generations was significant (p < 0.001).

Among the 92 pairs, only 5 (5.4%) first-generation members were diagnosed earlier (by 1, 5, 7, 9, and 16 years, respectively) compared with 87 (94.6%) second-generation members who showed earlier onset (anticipation) by a median of 28 years (range, 1 – 63 years). Of these 87 patients, 72 had anticipation of more than 15 years.

To address ascertainment bias, 41 pairs in which diagnosis occurred after age 25 years were re-analyzed; the results showed the same trend (**Table 5**). **Supplementary Table 1** provides details of all individual cases for future reference.

We also examined sex distribution across generations (“92-pair analysis”). In generation 1, male patients vs. female patients accounted for 63% and 37%, respectively, and in generation 2, 59.8% and 40.2% (p = 0.76). Although the proportion of male patients was higher, the difference was not statistically significant. 

## Discussion

This study reports a comprehensive initiative toward detailed data collection of FM and FHM from the Middle East, aiming to serve as a resource for engaging patients and families in future genetic counseling and studies. This analysis was limited to patients with NHL and HL seen in the Adult Medical Oncology Department, as patients with other hematological malignancies were treated in the Hematology Department. Our data showed that FHM has a substantial magnitude, necessitating comprehensive data capture strategies.

Being the first study of its kind regionally and internationally because of its different patient population, we encountered many challenges and learned valuable lessons (12). Gathering information about large families and time limitations in routine clinics was a real challenge. Although 1,274 patients answered the FM-CRF, only 32%–42% completed the details of their first-, second-, and third-degree relatives. With improved interview techniques, FM-CRF completion may reach 80%–90% with nurse or clinical coordinator follow-up. This is necessary, as younger patients often rely on their parents.

Confirming patients and family members treated at our institution by name was not easy, as the National Identity Document is in Arabic, and English spellings were entered either by hospital staff or as provided in the referral report. Even for many common first and last names, observing four to eight spelling variations was not uncommon. Similar limitations in reported diagnoses, as in our setting, were noted by Chang et al. (19) Many relatives were also not comfortable sharing their information when contacted.

Furthermore, in the Middle East, tribal social lifestyles, multiple marriages, consanguineous marriages within the same tribe, and the presence of half-uncles, half-aunts, and half-cousins require a very meticulous, time-consuming, and custom-designed data collection approach to overcome these challenges for future analysis.

Of the 1,274 patients, 750 (58.9%) reported malignancy in their families, with FHM present in 254 (19.9%). Several studies have reported differences in the frequency of solid and hematological cancers in patients with HL and various types of NHL (1, 4, 9). The same analysis performed for NHL and HL was also applied to HL versus NLPHL and showed that the only statistical difference in frequency was among patients with family members with hematological malignancies: 16.8% of NHL patients versus 22.6% of HL patients (p = 0.01). HL versus NLPHL showed no difference.

Brown et al. (1) from the Dana-Farber Cancer Institute (Boston, USA) reported on 1,948 patients with lymphoma seen from November 2004 to February 2007. In their cohort, 55.4% reported a first-degree relative with cancer and 65% reported a first- or second-degree relative with cancer. They also observed differences in the frequency of malignancy among patients: CLL/SLL, 70.1%; NHL, 64%; and HL, 61.2% (p = 0.02). In addition, 9.4% reported a first-degree and 13.6% a first- or second-degree relative with lymphoid malignancy.

Jones et al. (9) from British Columbia reported 140 families with lymphoid cancers and identified 353 lymphoid malignancies across first- to fourth-degree relatives. However, no information on unaffected family members was provided.

Chang et al. (4) reported the frequency of malignancies in first-degree relatives using the Swedish Multi-Generation Registry in 1,506 lymphoma patients. They found cancers in 698 (46.3%) and hematological cancers in 109 (7.2%). For 1,229 controls, the frequency was 545 (44%) and 52 (4%), respectively. They concluded that a history of hematopoietic malignancy in any first-degree relative was associated with an increased risk of all NHL (odds ratio = 1.8, 95% CI: 1.2 – 2.5).

Linabery et al. (10) from the Children’s Oncology Group, studied first- and second-degree relatives of 517 pediatric HL patients (≤14 years at diagnosis). They reported solid cancers in 290 (56%) and hematological malignancies in 43 (8.3%). For 783 controls, the frequency was 402 (51.3%) and 61 (7.8%), respectively. HL was associated with a positive family history (HR 5 1.20, 95% CI: 1.06 – 1.36).

We observed an apparent anticipation phenomenon in our patients: 95% showed anticipation, and the difference in median age at diagnosis between the first (58 years) and second (24 years) generations was 34 years (p < 0.001). These results are consistent with other studies reporting anticipation of 9–25 years (7, 8, 10, 13–15, 17, 18, 20). Daugherty et al. (16), using Swedish Registry data, failed to observe anticipation after comprehensive data analysis. In contrast, Shugart et al (8) reported evidence of anticipation. These findings suggest that the impact of various variables at different times may affect the observation of anticipation.

We also tested anticipation across different relationships (parent–child, grandparent–grandchild, and uncle/aunt–nephew/niece), and in all cases the results were significant. Similarly, for different pathologies (NHL, HL, and other hematological malignancies), anticipation was significant. To exclude ascertainment bias, we repeated the anticipation analysis in patients diagnosed after age 25 years, and the results were consistent with other studies after adjusting for ascertainment bias (7, 9, 14, 17) Earlier studies that included ascertainment bias often involved patients treated in the 1970s and 1980s, when nitrogen mustard–based regimens (for HL) or cyclophosphamide-based chemotherapy (for NHL and HL) of up to eight cycles were used. These treatments were associated with a higher incidence of infertility, an issue of much lower magnitude today.

Jones et al. (14) thoroughly evaluated the factors influencing ascertainment bias using British Columbia Cancer Registry data (Canada).

Only a few reports have investigated FM in patients with NLPHL (12, 21–25) with the largest data set from the Finnish Registry. They evaluated 692 patients and 4,280 first-degree relatives, and the standardized incidence ratio was 19% in first-degree relatives (24). Giles et al. identified 13 potential families with NHL and found that the overall risk for first-degree relatives of an affected individual was 3.15 – 3.61 (26).

Consanguineous marriages are a common practice in the Middle East. It was difficult to assess the frequency in this study, as the data were either missing or the patient was single (76%). For parents, 74% of data were missing. Among those who provided information, 38.2% of patients and 35% of parents reported consanguineous marriages. These figures are consistent with published data for the region (40%–55%). Importantly, this study shows that the current generation has a similar frequency of consanguineous marriages as the previous generation (27, 28).

Although our study is the first from the region and reports the largest data set, it has several limitations. The patients were seen at a tertiary care facility over a long period. Approximately 38% of hematological malignancies were unconfirmed, as they were self-reported. Follow-up with the FH-CRF was suboptimal, as it was not planned with allocated resources. Spelling issues limited confirmation of cases from the National Cancer Registry. Many patients did not provide information about their family members. These limitations could be minimized if the familial or related questionnaire were administered early in the course of patient visits, with dedicated research coordinators available for follow-up.

Unlike other studies, these data represent NHL and HL only, with negligible numbers of CLL/SLL, which were more substantial in other similar reports. We do not have data from national resources on the incidence and frequency of familial malignancies in patients with malignancies.

The understanding of genetic abnormalities, DNA repair mechanisms, and cell signaling pathways, along with their emerging interactions in low-grade lymphomas, is evolving (29). These studies are exploring opportunities to identify genetic-level interactions. It would be valuable to explore, in patients with anticipation, whether early disease manifestation is due to specific genetic interactions. Pooling data from patients with low-grade lymphomas and anticipation may help to explore this further.

Despite its limitations, this study provided valuable insights into FM and FHM in Middle Eastern countries, including interviewing techniques, resource allocation, methods for confirming malignancies, and the use and limitations of national cancer registries. Our large data set will help implement the main project of genetic counseling. This project aims to explain and educate accessible patients/family members about the importance of FM and to engage them in genetic studies.

## Data Availability

The original contributions presented in the study are included in the article/**Supplementary Material**. Further inquiries can be directed to the corresponding author.
