# Supplementary material for: Risk of hematological malignancies and anticipation in the families of patients with non-Hodgkin and Hodgkin lymphoma
Source: Front Oncol. 2025 Sep 9;15:1639819. doi: 10.3389/fonc.2025.1639819 (PMC12454086; doi:10.3389/fonc.2025.1639819)
Supplement: Supplementary file 1 [file DataSheet1.pdf]

Table 1. Supplemental. Anticipation details in ninety-two pairs									
		Patients				Family members			Anticipation age early /- late
Pair		Family No	Relationship	Age at diagnosis	Diagnosis	Relationship	Age at diagnosis	Diagnosis	
1	Female	1	Mother	78	NHL-NOS	Son	15	Leukemia-ALL	63
2	Female	1.1	Grand Mother	78	NHL-NOS	Grand Daughter	18	NHL DLBCL	60
3	Female	2	Nephew	30	DLBCL	Aunt	57	Multiple Myeloma	27
4	Female	2.1	Nephew	30	DLBCL	Aunt	63	NHL CLL SLL	33
5	Male	3	Father	65	DLBCL	Daughter	15	NHL DLBCL	50
6	Male	3.1	Father	65	DLBCL	Son	46	cHL	19
7	Male	4	Uncle	20	DLBCL	Nephew	18	Leukemia-ALL	2
8	Male	4.1	Nephew	20	DLBCL	Aunt	30	NHL DLBCL	10
9	Female	5	Daughter	33	DLBCL	Father	42	NHL CLL SLL	9
10	Female	5.1	Niece	33	DLBCL	Aunt	17	cHL	-16
11	Female	6	Niece	24	DLBCL	Uncle	47	Leukemia-AML	23
12	Female	6.1	Niece	24	DLBCL	Uncle	19	NHL DLBCL	-5
13	Male	7	Son	38	MZL	Father	95	NHL DLBCL	57

14	Female	8	Uncle	64	NHL-NOS	Nephew	12	Leukemia-NOS	52
15	Female	9	Grand Daughter	23	DLBCL	Grand Mother	73	NHL Low grade	50
16	Male	10	Grand Son	30	DLBCL	Grand Father	80	Leukemia-NOS	50
17	Female	11	Daughter	24	DLBCL	Father	72	NHL DLBCL	48
18	Male	12	Father	79	FCC	Daughter	34	NHL Burkitts	45
19	Female	13	Daughter	39	DLBCL	Father	79	NHL DLBCL	40
20	Female	14	Daughter	34	DLBCL	Father	74	NHL DLBCL	40
21	Female	15	Grand Mother	53	DLBCL	Grand Daughter	14	BLOOD NOS	39
22	Female	16	Grand Mother	54	MZL	Grand Daughter	17	Leukemia-AML	37
23	Male	17	Uncle	68	DLBCL	Nephew	31	NHL NOS	37
24	Male	18	Uncle	43	ALCL	Niece	6	BLOOD NOS	37
25	Male	19	Nephew	31	DLBCL	Aunt	60	NHL NOS	29
26	Male	20	Son	52	DLBCL	Mother	80	NHL NOS	28
27	Female	21	Mother	55	MZL	Son	30	HL	25
28	Female	22	Daughter	34	DLBCL	Father	59	NHL NOS	25
29	Female	23	Daughter	51	DLBCL	Father	76	Leukemia-CLL	25
30	Male	24	Son	36	CLL/SLL	Father	60	Leukemia-CLL	24

31	Female	25	Mother	57	FCC	Son	34	NHL DLBCL	23
32	Male	26	Nephew	15	DLBCL	Uncle	37	NHL/HL NOS	22
33	Female	27	Aunt	48	NHL-NOS	Nephew	31	NHL/HL NOS	17
34	Female	28	Daughter	63	ALCL	Mother	80	NHL/HL NOS	17
35	Male	29	Uncle	33	DLBCL	Nephew	17	Leukemia-Aplastic	16
36	Female	30	Daughter	66	MALT	Mother	77	NHL DLBCL	11
37	Male	31	Nephew	45	DLBCL	Uncle	56	Leukemia-AML	11
38	Male	32	Nephew	73	DLBCL	Uncle	77	Leukemia-NOS	4
39	Male	33	Son	34	cHL	Father	65	BLOOD NOS	31
40		33.1	Uncle	34	cHL	Nephew	15	NHL Burkitts	19
41	Female	34	Niece	35	NLPHL	Aunt	24	NHL DLBCL	-9
42	Female	35	Niece	29	cHL	Aunt	22	HL	-7
43	Male	36	Nephew	13	cHL	Grand Father	71	NHL Burkitts	48
44	Male	36.1	Grand Son	13	cHL	Uncle	16	Leukemia-ALL	3
45	Female	37	Uncle	59	cHL	Niece	25	NHL NOS	34
46	Female	37.1	Uncle	59	cHL	Niece	60	Leukemia-NOS	-1
47	Female	38	Grand Daughter	25	cHL	Grand Father	85	Leukemia-NOS	60

48	Male	39	Grand Son	29	NLPHL	Grand Mother	87	NHL/HL NOS	58
49	Female	40	Grand Daughter	19	cHL	Grand Father	77	NHL DLBCL	58
50	Male	41	Son	16	cHL	Father	69	NHL Low grade	53
51	Male	42	Grand Son	22	NLPHL	Grand Father	72	NHL/HL NOS	50
52	Male	43	Son	10	NLPHL	Father	38	NHL NOS	48
53	Male	44	Grand Son	22	cHL	Grand Father	70	NHL DLBCL	48
54	Male	45	Nephew	17	cHL	Aunt	63	NHL DLBCL	46
55	Male	46	Father	46	cHL	Daughter	6	Leukemia-NOS	40
56	Male	47	Father	53	cHL	Son	11	HL	45
57	Female	48	Grand Daughter	24	cHL	Grand Mother	69	Leukemia-NOS	45
58	Male	49	Son	15	cHL	Mother	60	Multiple Myeloma	45
59	Male	50	Nephew	14	cHL	Aunt	59	HL	45
60	Male	51	Grand Son	18	NLPHL	Grand Father	62	NHL DLBCL	44
61	Female	52	Niece	28	cHL	Uncle	70	Leukemia-AML	42
62	Female	53	Daughter	23	cHL	Father	64	NHL Low grade	41
63	Female	54	Daughter	17	cHL	Father	54	NHL Burkitts	37
64	Male	55	Father	54	cHL	Son	18	HL	36

65	Male	56	Father	81	cHL	son	45	HL	36
66	Male	57	Nephew	24	cHL	Aunt	60	NHL DLBCL	36
67	Male	58	Father	53	NLPHL	Son	19	NHL CLL SLL	34
68	Male	59	Son	30	cHL	Father	62	NHL NOS	32
69	Male	60	Son	34	cHL	Mother	65	NHL DLBCL	31
70	Male	61	Nephew	32	NLPHL	Uncle	60	Leukemia-NOS	28
71	Female	62	Niece	26	NLPHL	Uncle	53	NHL/HL NOS	27
72	Male	63	Nephew	27	NLPHL	Uncle	54	NHL CLL SLL	27
73	Male	64	Son	26	cHL	Father	53	NHL T ALCL	27
74	Male	65	Father	53	cHL	Daughter	26	NHL NOS	27
75	Female	66	Mother	41	cHL	Son	16	HL	25
76	Male	67	Uncle	35	cHL	Nephew	10	Leukemia-NOS	25
77	Female	68	Daughter	31	cHL	Father	56	Leukemia-CLL	25
78	Female	69	Daughter	36	cHL	Mother	60	NHL DLBCL	24
79	Female	70	Aunt	32	cHL	Nephew	10	Leukemia-NOS	22
80	Female	71	Aunt	42	cHL	Niece	21	Leukemia-NOS	21
81	Male	72	Son	53	NLPHL	Mother	73	NHL DLBCL	20

82	Female	73	Daughter	30	NLPHL	Father	59	Leukemia-NOS	19
83	Male	74	Nephew	15	NLPHL	Uncle	32	Leukemia-AML	17
84	Female	75	Niece	20	cHL	Uncle	35	Leukemia-NOS	15
85	Male	76	Nephew	33	NLPHL	Uncle	47	HL NLP	14
86	Male	77	Nephew	12	cHL	Aunt	26	cHL	14
87	Male	78	Son	40	cHL	Father	50	Leukemia-NOS	10
88	Male	79	Nephew	17	cHL	Aunt	27	Leukemia-AML	10
89	Female	80	Aunt	17	cHL	Niece	13	Leukemia-NOS	4
90	Female	81	Aunt	26	cHL	Nephew	22	Leukemia-NOS	4
91	Male	82	Uncle	13	cHL	Nephew	11	Leukemia-NOS	2
92	Male	83	Nephew	15	NLPHL	Uncle	16	NHL NOS	1
